# Hypoxia-Targeting Fluorescent Nanobodies for Optical Molecular Imaging of Pre-Invasive Breast Cancer

**DOI:** 10.1007/s11307-015-0909-6

**Published:** 2015-11-20

**Authors:** Aram S. A. van Brussel, Arthur Adams, Sabrina Oliveira, Bram Dorresteijn, Mohamed El Khattabi, Jeroen F. Vermeulen, Elsken van der Wall, Willem P. Th. M. Mali, Patrick W. B. Derksen, Paul J. van Diest, Paul M. P. van Bergen en Henegouwen

**Affiliations:** Division of Cell Biology, Department of Biology, Science Faculty, Utrecht University, Padualaan 8, 3584 CH Utrecht, The Netherlands; Department of Pathology, University Medical Center Utrecht, Utrecht, The Netherlands; Department of Radiology, University Medical Center Utrecht, Utrecht, The Netherlands; Division of Internal Medicine and Dermatology, University Medical Center Utrecht, Utrecht, The Netherlands; QVQ BV, Utrecht, The Netherlands

**Keywords:** Carbonic anhydrase IX, Nanobody, VHH, Optical imaging, Molecular fluorescence pathology, Breast cancer

## Abstract

**Purpose:**

The aim of this work was to develop a CAIX-specific nanobody conjugated to IRDye800CW for molecular imaging of pre-invasive breast cancer.

**Procedures:**

CAIX-specific nanobodies were selected using a modified phage display technology, conjugated site-specifically to IRDye800CW and evaluated in a xenograft breast cancer mouse model using ductal carcinoma in situ cells (DCIS).

**Results:**

Specific anti-CAIX nanobodies were obtained. Administration of a CAIX-specific nanobody into mice with DCIS xenografts overexpressing CAIX showed after 2 h a mean tumor-to-normal tissue ratio (TNR) of 4.3 ± 0.6, compared to a TNR of 1.4 ± 0.2 in mice injected with the negative control nanobody R2-IR. In DCIS mice, a TNR of 1.8 ± 0.1 was obtained. Biodistribution studies demonstrated an uptake of 14.0 ± 1.1 %I.D./g in DCIS + CAIX tumors, 4.6 ± 0.8 %I.D./g in DCIS tumors, while 2.0 ± 0.2 %I.D./g was obtained with R2-IR.

**Conclusions:**

These results demonstrate the successful generation of a CAIX-specific nanobody-IRDye800CW conjugate that can be used for rapid imaging of (pre-)invasive breast cancer.

**Electronic supplementary material:**

The online version of this article (doi:10.1007/s11307-015-0909-6) contains supplementary material, which is available to authorized users.

## Introduction

Molecular imaging modalities such as positron emission tomography (PET), single photon emission computed tomography (SPECT), and optical imaging use antibodies or antibody-fragments to specifically track molecules or cells [1]. The use of a targeting moiety, specific to antigens present on tumor cells, results in higher contrast images compared to imaging strategies with non-targeted contrast agents [2–6]. Molecular imaging with fluorescent tracers (optical molecular imaging) has recently gained more interest [7–9], since it does not require expensive imaging equipment or protective measures due to the absence of ionizing radiation. As a result of the limited penetration of light into tissue, optical imaging is especially suitable for imaging of superficial tumors, such as head and neck cancer [10]. Moreover, optical imaging can be of help during tumor resection and could reduce unneeded surgical procedures. In addition, optical imaging is suitable for characterization of histological biopsies and/or surgical specimens, an approach we previously indicated as molecular fluorescence pathology [11].

Hypoxia is a condition that is present in the majority of solid tumors and normally absent in healthy tissue [12–14]. Several studies have demonstrated molecular imaging with radiolabeled hypoxia-specific probes, such as 2-nitroimidazoles, [^18^F]fluoromisonidazole, and Copper(II)-diAcetyl-bis(*N*^4^-methylThioSe-Micarbazone) ([^62^Cu]ATSM) [15, 16]. These studies showed only moderate tumor contrast mainly because of non-specific probe uptake. Higher tumor-specificity might be obtained by directly targeting proteins that are upregulated under hypoxic conditions, such as carbonic anhydrase IX (CAIX). CAIX expression is under control of hypoxia-inducible factor 1α (HIF-1α), a transcription factor that is stabilized under hypoxic conditions [17, 18]. We selected CAIX as target for molecular imaging, as it is one of the most tumor-specific membrane-bound proteins expressed in hypoxic tumors. Although CAIX expression might not reflect acute hypoxia because of a half-life of a few days, it is a marker of chronic hypoxia in tumors. CAIX can therefore be considered as a suitable marker that can be used to discriminate cancer from non-cancerous tissues [19–21].

In a previous study, we presented the successful optical imaging of pre-invasive cancer of the breast (ductal carcinoma in situ, DCIS) with a CAIX-specific conventional antibody (MabCAIX) [11]. Slow clearance of the antibody resulted in suboptimal contrast during the first 24 h post injection, and optimal tumor-to-normal tissue ratios (TNR) were obtained 72 h after probe administration. More rapid imaging would result in lower costs as less logistical procedures are needed, and it would be more convenient for the patient and healthcare workers. Faster clearance and subsequent faster image acquisition with higher contrast can be obtained using probes with molecular sizes that are below the renal glomerular filtration threshold of about 50 kDa, as recently demonstrated for the CAIX-specific tracer HS680, which consists of a CAIX inhibitor conjugated to the fluorescent dye Vivotag680 [22].

Promising probes for rapid molecular imaging are nanobodies or VHHs (variable domain of heavy chain antibodies), which are antibody-fragments derived from heavy chain antibodies that naturally occur in camelids [23]. Compared to conventional antibodies, nanobodies possess several advantageous properties. Firstly, the molecular weight of nanobodies is ten times lower (15 kDa vs. 150 kDa), which results in rapid tumor accumulation while having short elimination half-life in the bloodstream, which together lead to good contrast at early time points after administration [24]. Secondly, nanobodies can be selected to bind with high affinity to their target, and this high affinity is essential for accumulation in the tumor [24]. Thirdly, nanobodies can easily be produced in different organisms such as bacteria, yeast and mammalian cells, and they are more stable than other antibody-fragments [25]. Finally, nanobodies are, thus far, known to be non-immunogenic [25]. We and others have previously shown that nanobodies can successfully be used for rapid molecular imaging of both epidermal growth factor receptor (EGFR) and human epidermal growth factor receptor 2 (HER2) [9, 24, 26, 27].

In the current study, we present phage display selections for nanobodies that specifically bind to the ectodomain of CAIX. CAIX-specific nanobodies were selected from a library derived from llamas immunized with hypoxic HeLa cells. A second-generation library was produced on basis of polymerase chain reaction (PCR) using complementarity determining region 3 (CDR3) sequences from two initial anti-CAIX nanobodies, and from this library, high-affinity binders with specificity for CAIX were obtained. To avoid affinity loss because of random conjugation, the nanobodies were site-directedly conjugated to IRDye800CW and evaluated by optical molecular imaging of CAIX overexpressing or endogenously expressing tumors in an orthotopic mouse model of DCIS. Our data supports application of anti-CAIX nanobodies for pre-, intra-, and postoperative optical imaging of (pre-invasive) breast cancer and holds promise for broader applications such as clinical PET or SPECT imaging.

## Materials and Methods

### Phage Display Selections

Maxisorp plates were coated with 1.00, 0.50, 0.10, 0.05, and 0.00 μg recombinant CAIX (R&D systems, Minneapolis, USA). Phages were produced from *E.coli* TG1 harboring the library after infection with helper phage VSCM13 (Strategene, Agilent Technologies Netherlands B.V., Amstelveen, The Netherlands) and incubating overnight while shaking at 37 °C in medium containing ampicillin (100 μg/ml) and kanamycin (25 μg/ml). The next day, maxisorp wells were washed three times with PBS and blocked with 4 % marvel in PBS. Phages were precipitated by adding 2 % polyethylene glycol (PEG) and 250 mM NaCl for 30 min on ice. After spinning down and resuspending the pellet in ice cold PBS, PEG precipitation was repeated twice. After resuspension, phages were incubated in the blocked maxisorp wells for 2 h at room temperature, while shaking. Non-specific phages were removed by washing twenty times with PBS containing 0.05 % Tween, every fifth time shaking for 10 min. Bound phages were eluted by trypsin digestion (1.0 mg/ml) for 20 min and infection of an *E.coli* culture in the exponential phase of the growth for 30 min after adding trypsin inhibitor. After infection, phages were titrated, spotted on agar plates (containing 100 μg/ml ampicillin and 2 % glucose) to calculate the number of bound phages. Subsequently, the infections were grown overnight in 2TY medium containing 100 μg/ml ampicillin and 2 % glucose, shaking at 37 °C. The next day, the overnight culture was used for phage production. On the third day, a second round of selections was performed by incubating output phages from the first round of selections in wells coated with a concentration range of 0.01 to 1.00 μg recombinant CAIX.

### Nanobody Production and Purification

Nanobodies were re-cloned from the pUR8100 phagemid vector into the pQVQ72 expression vector (kindly provided by QVQ BV, Utrecht, The Netherlands), which introduces a C-terminal cysteine, flanked by a Flag-tag to enable site-directed conjugation of IRDye800CW-Maleimide (LI-COR Biosciences, Lincoln, NE) (see electronic supplementary material (ESM) for more details).

### Generation of Family-Specific Phage sub-Library (“Family Approach”)

Based on the sequence of two anti-CAIX nanobodies, two family-specific sub-libraries were made following the procedure previously described [28], with a few modifications. A unique degenerate reverse primer extending into the entire CDR3 loop region was designed and used in conjunction with the plasmid-based primer (M13 rev) to PCR VHH gene fragments with the same CDR3 present in the library. Amplification was carried out with Phusion High-Fidelity DNA Polymerase (Thermo Fisher Scientific, Landsmeer, The Netherlands), and a 350-bp band was excised after separation on an agarose gel. Following restriction enzyme digestion with BstEII and SfiI and gel purification, the digested DNA fragments were ligated into the phagemid vector pUR8100 for display on filamentous bacteriophage and transferred to *E. coli* TG1 competent cells by electroporation. The resulting two family libraries were used for phage display selections as described above.

### Conjugation of IRDye800CW to CAIX Nanobodies

Before IRDye800CW labeling, nanobodies were reduced by adding 70-fold molar excess of Tris (2-CarboxyEthyl) Phosphine hydrochloride (TCEP). IRDye800CW-Maleimide (further referred to as IR; LI-COR) was conjugated to nanobodies following manufacturer recommendations (see ESM for more details).

## Results

### Immunization, Library Construction, Phage Display Selections, and Screening

**Fig. 1:**
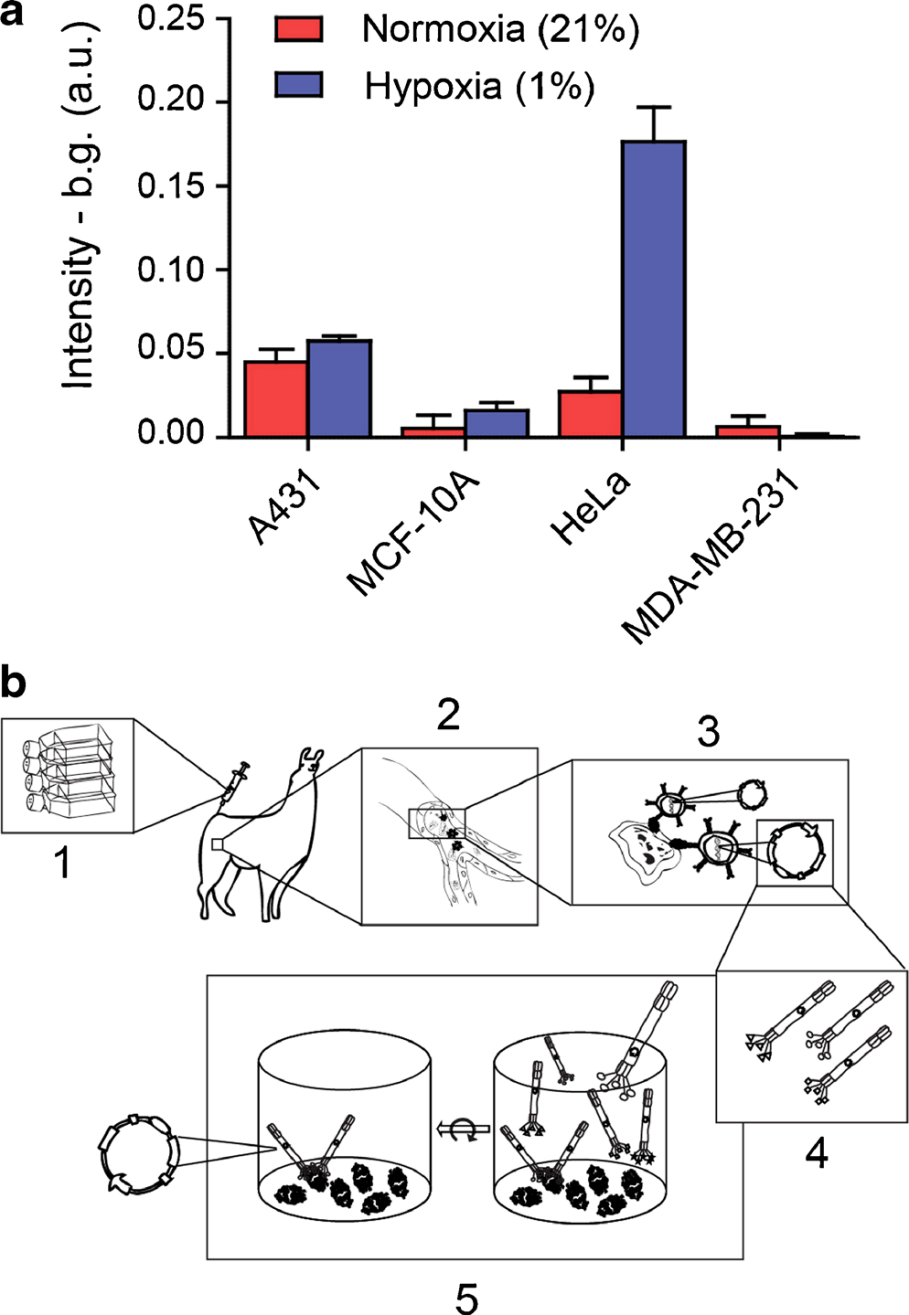
Hypoxic HeLa cells can be used for generation of a hypoxia-specific phage library. **a** Indicated cells were grown under normoxic (21 % O_2_) (*red*) and hypoxic (1 % O_2_) (*blue*) conditions for 24 h, and CAIX levels were determined by a cell-based ELISA as described in Materials and Methods in the ESM. **b** Workflow of phage display selections: 1. Llamas are immunized with hypoxic HeLa cells; 2. During the immune response peripheral B-lymphocytes generate CAIX-specific heavy chain-only antibodies; 3. peripheral B-lymphocytes are isolated and RNA is extracted. After reverse transcriptase PCR, antibody specific DNA is ligated into a phagemid vector; 4. Phages expressing nanobodies at their surface are produced in *E. coli* bacteria; 5. Two rounds of phage display selections are performed in a 96-wells format coated with recombinant CAIX.

To enable llama immunization with cells showing sufficient levels of CAIX expression, we first evaluated CAIX expression levels of several cell lines cultured in vitro under normoxic and hypoxic conditions [29]. Cells were grown for 24 h at 1 % O_2_, and the CAIX expression was analyzed by cell-based enzyme-linked immunosorbent assay (ELISA). HeLa cells showed the highest CAIX upregulation and no CAIX expression was observed in the control cell line (Fig. 1a). As described in the ESM, two llamas were immunized with hypoxic HeLa cells, which express native CAIX, and pre- and post-immunization sera were used to follow the development of an immune response against CAIX. A VHH phagemid library was prepared as described previously [30], and using this library, various phage display selections were performed using either directly coated recombinant CAIX, captured recombinant CAIX or hypoxic HeLa cells with or without specific elution using anti-CAIX mAb (Fig. 1b). After two rounds of biopanning, the binding of nanobodies provided with a his- and myc-tag, was analyzed in vitro using CAIX-overexpressing or CAIX negative MCF10DCIS cells grown under normoxic conditions [11]. From these screenings, two CAIX-specific nanobodies were isolated, indicated as CAIX1 and CAIX4.

### Nanobody Characterization and Generation of a CAIX Family-Specific Phage Library

**Fig. 2:**
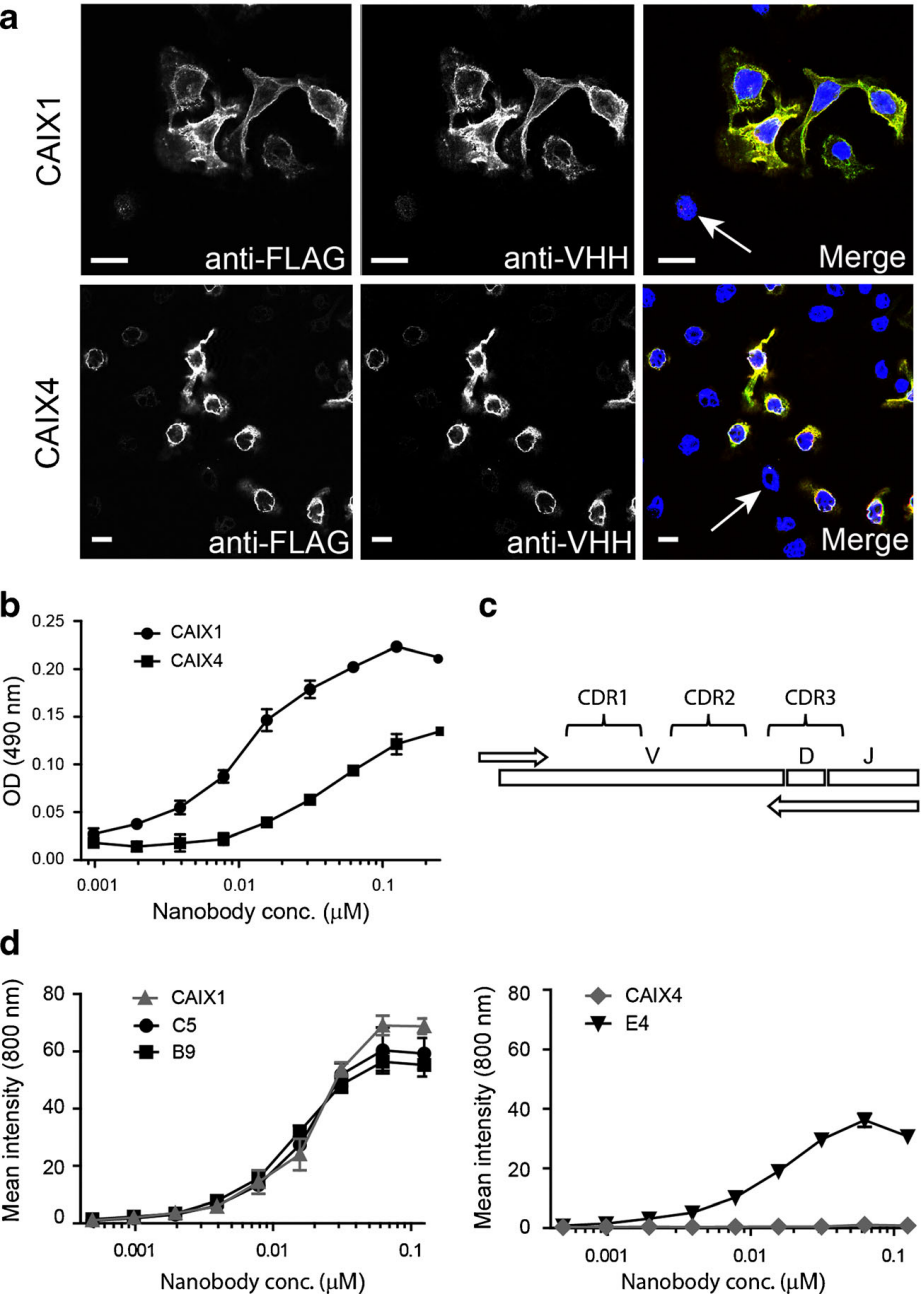
CAIX1 is a high-affinity CAIX-specific nanobody, and CAIX1 DNA can serve as template for the family approach. **a** Co-cultures of CAIX-FLAG expressing and CAIX negative cells were incubated with either CAIX1 or CAIX4 nanobody. Nuclei were stained with DAPI (*blue*). Bound nanobodies were detected with Alexa-488 (*green*) and CAIX-FLAG with Alexa-555 (*red*). The overlay is shown in the *right panels. Arrows* indicate cells without CAIX expression. Scale bar: 10 μm. **b** DCIS + CAIX cells were incubated with a dilution series of CAIX1 and CAIX4 nanobodies, which were detected using anti-VHH antibodies and peroxidase conjugated secondary antibodies. The *y*-axis shows intensity of peroxidase substrate. **c** Library DNA is used as template in a PCR reaction with family-specific reverse primers covering CDR3. The PCR product is ligated in a phagemid vector resulting in a family-specific phage library. **d** DCIS + CAIX cells were incubated with a dilution series of nanobody conjugated to IRDye800CW: CAIX1 (*left*, *gray*), CAIX4 (*right*, *gray*) and the nanobodies derived from the family-specific library (*black*) based on CDR3 of either CAIX1 (C5 and B9) or CAIX4 (E4). The *y*-axis shows fluorescence intensity measured by the Odyssey system.

To verify the specificity of the CAIX1 and CAIX4 nanobodies, immunofluorescence studies were performed using co-cultures of CAIX-overexpressing (DCIS + CAIX) and CAIX negative (DCIS) MCF10DCIS cells. Cells were incubated with CAIX1 or CAIX4 nanobodies, which were detected with antibodies directed against VHHs (Fig. 2a, green). Expression of FLAG-CAIX was verified using anti-FLAG antibodies (Fig. 2a, red). The overlay shows that both nanobodies are binding specifically to the DCIS + CAIX cells. CAIX negative cells did not show any nanobody binding (Fig. 2a, arrows). Subsequently, binding experiments were performed (as described in the ESM) to determine apparent affinities (K_D_) of the unconjugated nanobodies for CAIX, which were 11 nM for CAIX1 and 45 nM for CAIX4 (Fig. 2b).

To obtain anti-CAIX nanobodies with higher affinities, a new second-generation family-specific library was made as previously described by Koh et al. [28]. Primers were designed based on the N-terminal framework sequence and on the CDR3 sequence of CAIX1 or 4, and a PCR was performed using the original library as template (Fig. 2c). A novel CAIX library was constructed by ligating the PCR products into a phagemid vector. With this novel CAIX library, phage display selections were performed using recombinant CAIX, resulting in the isolation of three additional anti-CAIX nanobodies indicated as C5, B9 (both based on CAIX1), and E4 (based on CAIX4). Affinity determination of the C5 and B9 nanobodies showed an improvement in affinity from the original 11 nM (CAIX1) to 6 nM (C5) and 7 nM (B9). Affinity also improved with the CAIX4-based family approach: from 45 nM for the CAIX4 nanobody to 2 nM, which was the affinity of the novel anti-CAIX nanobody E4.

### Characterization of IR-Conjugated CAIX Nanobodies

The four CAIX-specific nanobodies were conjugated site-directedly via a C-terminal cysteine residue (as described previously [9]), as random conjugation could considerably reduce the affinity of the nanobodies. After conjugation with maleimide-IRDye800CW, the degree of labeling was approximately 0.6 for all nanobodies. The binding affinity of the nanobodies (*K*_D_) was determined using DCIS + CAIX cells. In pilot studies with CAIX negative MCF10DCIS, signals did not exceed background (data not shown). The binding affinities were 19 nM (CAIX1-IR), 17 nM (C5-IR), 13 nM (B9-IR), and 8 nM (E4-IR) (Fig. 2d). Despite the site-directed dye conjugation, this labeling procedure reduced the binding affinity and had even a detrimental effect on the affinity of CAIX4-IR. The *B*_max_ of all nanobodies was comparable to CAIX1-IR (~80 a.u.), except for E4-IR, which had a lower *B*_max_ (~40 a.u.; Fig. 2d). We selected B9-IR as lead nanobody for further in vivo studies because of the best binding affinity (13 nM) in combination with a high *B*_max_.

The specificity of the B9 nanobody for CAIX was confirmed in two ways. First, we compared immunofluorescence of DCIS cells with the same cells ectopically expressing CAIX (DCIS + CAIX). A clear difference was observed, i.e., more binding was observed for the cell line with the highest expression of CAIX (Fig. 1S). Subsequently, we incubated DCIS + CAIX cells with a mixture of 2.5 nM B9 with 500 molar excess of the human recombinant CAIX ectodomain. No immunofluorescence was observed, demonstrating that the binding of the B9 nanobody was competed off by the presence of the CAIX ectodomain (Fig. 1S).

### In Vivo and Ex Vivo Optical Imaging

**Fig. 3:**
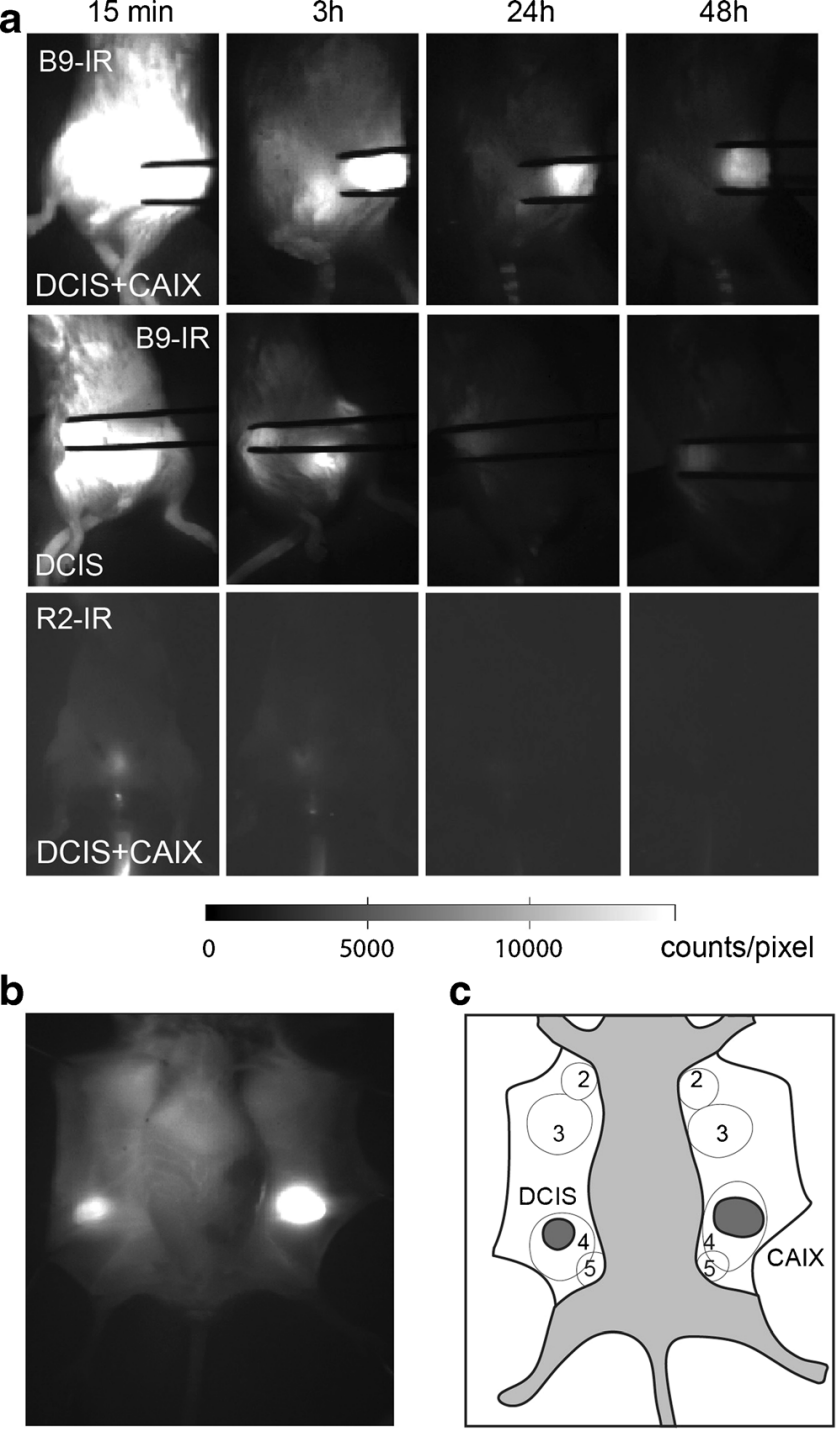
Detection of hypoxic pre-invasive breast tumors in vivo and intra-operatively using the B9-IR nanobody. **a** DCIS + CAIX and DCIS xenografts were imaged at several time points post injection of 50 μg B9-IR nanobody or 50 μg R2-IR. Tumors were held between tweezers. **b** Intra-operative imaging of DCIS and DCIS + CAIX tumors, 3 h post injection of B9-IR. **c** Schematic overview of mammary glands (2–5) and tumors as seen intra-operatively. DCIS + CAIX tumor indicated as “CAIX” in *dark gray*.

The feasibility of optical imaging with the B9-IR CAIX-specific nanobody was tested in a preclinical setting, with SCID/Beige mice that were orthotopically transplanted with MCF10DCIS cells expressing CAIX peri-necrotically (‘DCIS’ tumors) and MCF10DCIS cells stably expressing exogenous CAIX (“DCIS + CAIX” tumors). A non-relevant R2 nanobody was used as a negative control [9, 24]. Upon development of palpable tumors, mice were injected in the tail vein with 50 μg of the indicated nanobodies. Fluorescent probe distribution was visualized at several time points up to 48 h post injection using an imaging camera developed and approved for clinical use [31]. Already 2–3 h p.i., we could delineate both the DCIS + CAIX and DCIS tumors from the background non-invasively (Fig. 3a) and invasively (Fig. 3b, c).

**Fig. 4:**
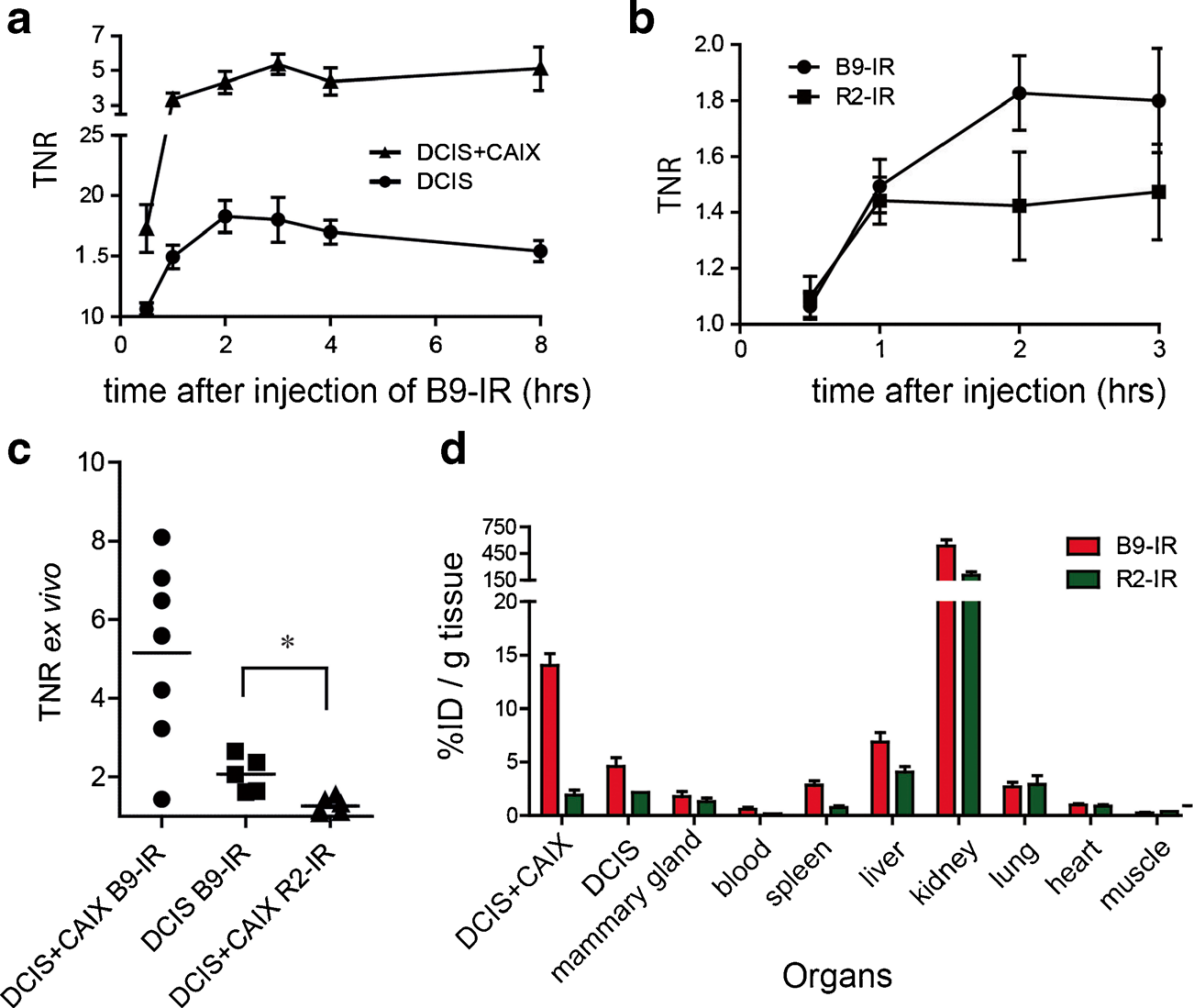
Optimal imaging with B9-IR nanobody 2 h post injection. **a** Mean TNR of CAIX-overexpressing tumors (DCIS + CAIX, *n* = 10), and DCIS tumors (*n* = 10) determined during the first 8-h post injection of B9-IR nanobody. *Error bars* represent SEM. **b** Mice xenografted with DCIS tumors were injected with 50 μl B9-IR (10 mice) or R2-IR (4 mice) non-relevant control nanobody, mean TNR values were determined at indicated time points. **c** DCIS + CAIX (7 mice) and DCIS (7 mice) tumors after injection with 50 μg B9-IR, and DCIS + CAIX tumors (*n* = 6) after injection with R2-IR non-relevant control nanobody. Single values of intra-operative TNRs were determined 3 h post injection. *Bar* represents the mean (*p = 0.04). **d** For a biodistribution assay, mice (*n* = 9) were injected with B9-IR or R2-IR non-relevant control nanobody. Tumors and organs were collected 3 h post injection. *Error bars* represent SEMs.

TNRs were calculated by dividing the fluorescent signal from the tumor by the signal from the hind leg for each time point up to 48 h. The mean in vivo DCIS + CAIX TNR increased in the first hour, until a plateau level was reached, which persisted for 8 h (Fig. 4a). Two hours after probe administration, a mean in vivo DCIS + CAIX TNR of 4.3 ± 0.6 (standard error of the mean (SEM), *n* = 8) was observed. At 2 h post injection, the mean DCIS TNR in mice injected with B9-IR was 1.8 ± 0.1 (*n* = 8), and mice injected with the R2-IR non-relevant control nanobody showed a TNR of 1.4 ± 0.2 (*n* = 4; *p* = 0.07; Fig. 4b). The TNR of 1.8 was sufficient to detect DCIS tumors with a diameter starting from 2 mm (data not shown).

After these studies, mice were sacrificed, and the skin was removed to enable ex vivo tumor imaging, simulating the surgical setting. The mean ex vivo or “intra-operative” TNR of CAIX-overexpressing tumors 3 h post injection was 5.2 ± 0.9, slightly higher than in the in vivo setting (Fig. 4c). The mean TNR of the DCIS tumors from mice injected with B9-IR or R2-IR was 2.1 ± 0.2 and 1.3 ± 0.1, respectively (*p* = 0.04). The maximum intra-operative TNR of the DCIS tumors obtained from mice injected with B9-IR or R2-IR was 2.7 and 1.5, respectively. These results show that DCIS + CAIX tumors can be detected both in vivo as well as under ex vivo conditions.

### Biodistribution

To quantify fluorescent probe signals from tumors and organs, we performed a biodistribution study, circumventing effects of scattering and quenching of the fluorescent signal [9, 32]. To determine the biodistribution of B9-IR and R2-IR, organs and tumors of nine mice were excised, weighted, and after processing, their fluorescent signals were determined. In the DCIS + CAIX tumors 14.0 ± 1.1 % of the injected dose of B9-IR per gram tissue (ID/g) was present, and 4.6 ± 0.8 %ID/g was found in DCIS tumors, which was significantly higher than for muscle or blood (0.2 ± 0.1 and 0.6 ± 0.2 %ID/g, respectively). In the DCIS + CAIX tumor 1.9 ± 0.5 %ID/g of the injected dose of the R2-IR negative control nanobody was found, which differs significantly from the B9 accumulation (*p* = 0.01). The difference between B9-IR and R2-IR for the DCIS tumor was not significant (4.6 ± 0.8 vs 2.0 ± 0.2 %ID/g; *p* = 0.2). Compared to other organs, kidney uptake of the nanobodies was high, which is due to kidney retention of the nanobodies, confirming the biodistribution assays performed previously [9, 24] (Fig. 4d).

### Imaging of Tumor Sections and Immunohistochemistry

**Fig. 5:**
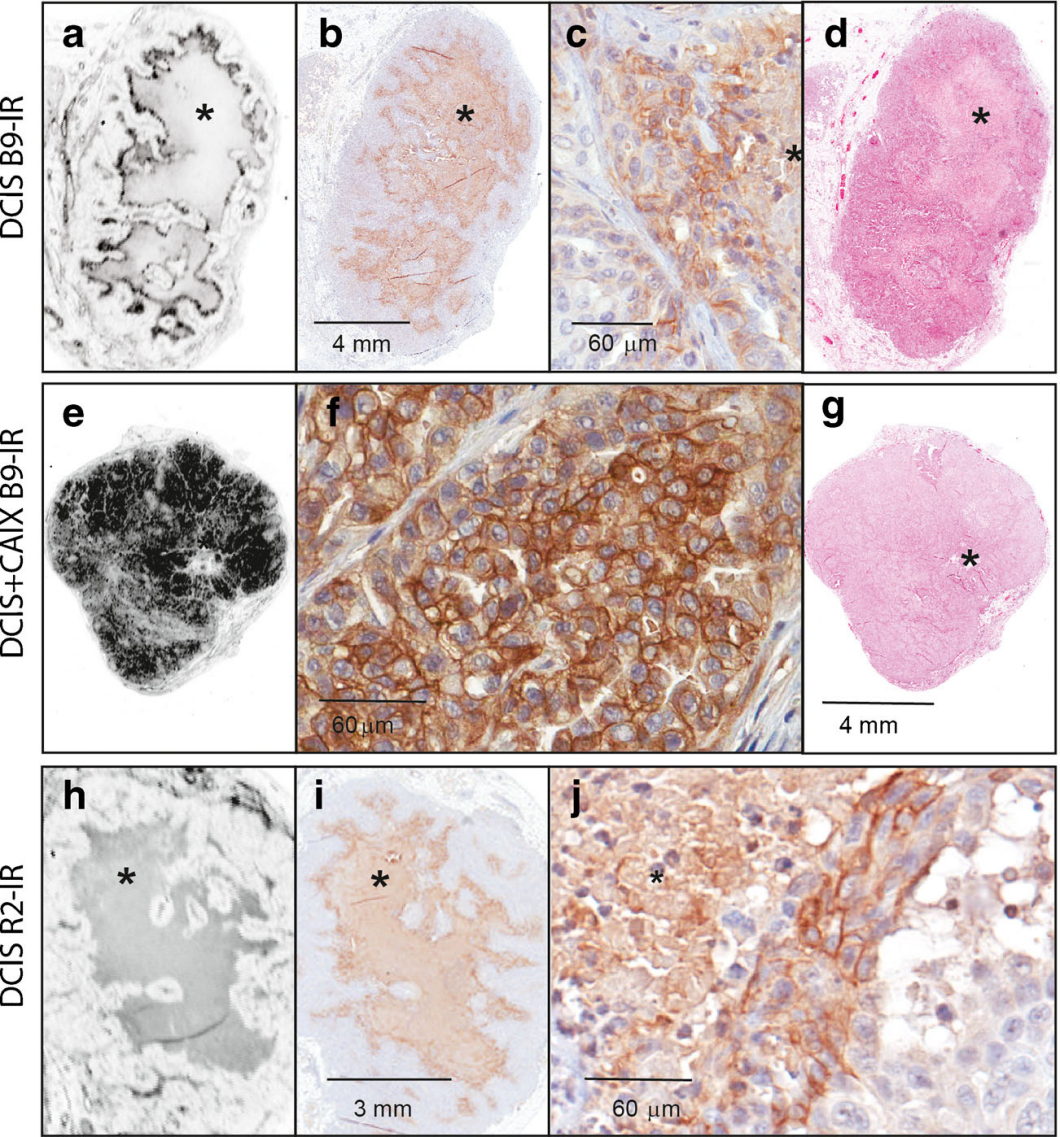
B9-IR binds to perinecrotic area in DCIS tumors. **a**–**d** DCIS tumor from a mouse injected with B9-IR nanobody, 3 h post injection. **e**–**g** DCIS + CAIX tumor from a mouse injected with B9-IR. h-j. DCIS tumor from a mouse injected with R2-IR (non-relevant control nanobody). **a** Fluorescence scan of DCIS tumor. **b** CAIX-IHC. **c** Magnification of perinecrotic area. **d** H&E staining of tumor section. **e** Fluorescence scan of DCIS + CAIX tumor. **f** Magnification of CAIX-IHC. **g** H&E staining of tumor section. **h** Fluorescence scan of DCIS tumor with central necrosis. **i** CAIX-IHC. **j** magnification of CAIX-IHC (*indicates necrotic area).

To investigate the binding of the anti-CAIX nanobodies to hypoxic areas in the tumors, tumors were collected 3 h post injection from mice injected with either B9-IR or R2-IR. Tumors were formalin fixed, paraffin embedded, and sections of the tumors were scanned with the Odyssey imaging system to detect IR fluorescence. In the DCIS tumors the anti-CAIX nanobody was clearly visible in the perinecrotic areas surrounding the necrotic area of the xenografts (Fig. 5a–d). DCIS + CAIX tumors showed a homogeneous fluorescent staining of the xenografts (Fig. 5e–g). B9-IR staining of the xenografts co-localized with the intra-tumoral membraneous distribution of CAIX, which was detected by immunohistochemistry using an anti-CAIX antibody. The non-relevant control nanobody R2-IR showed no uptake in tumor tissue (Fig. 5h–j). However, fluorescent sections together with H&E stained sections demonstrated the limited uptake of R2-IR in surrounding mouse mammary gland tissue.

## Discussion

For rapid molecular imaging of (pre-invasive) tumors in general and breast cancer in particular, targeting of tumor biomarkers using optical tracers or radiotracers is essential for contrast enhancement. Valid tumor markers are plasma membrane proteins that are specifically expressed in the tumor and not in surrounding tissue, such as HER2 [9]. However, HER2 is only expressed in 15–20 % of breast cancers; thus, the drawback of some of these markers is their expression in only a small percentage of breast cancers. As a more general marker for cancer, we selected CAIX, which is substantially upregulated under hypoxic conditions at the cell surface of many tumor types. Others have evaluated both CAIX inhibitors and CAIX antibodies for in vivo tumor imaging [11, 21, 22].

A novel and versatile targeting platform are nanobodies. In the current study, we have generated hypoxia-specific nanobodies that specifically bind to the hypoxia marker CAIX. These anti-CAIX nanobodies were selected by a phage display family approach using an immune sub-library in order to select for nanobodies against CAIX with the highest affinity possible. The success of this approach was demonstrated by the fact that affinities of these nanobodies were better than the affinities from nanobodies selected from the original immune library. Recently, a different study reported the selection of nanobodies binding to CAIX, which was shown to have a binding affinity (*K*_D_) of 23 μM [33]. As previously shown, nanobodies with an affinity of 1 μM did not sufficiently accumulate in the tumor, emphasizing the need for high-affinity binders [9, 34]. In this context, we have shown that the binding affinity can seriously drop after the conjugation of the nanobody to the tracer, in this case the fluorophore IRDye800CW. As this was also the case for the selected anti-CAIX nanobodies, the dye was conjugated via a C-terminal cysteine. The B9-IR nanobody was finally selected as lead product because of the excellent binding properties (*K*_D_ = 13 nM) in combination with high *B*_max_.

An orthotopic xenograft mouse model was used to validate tumor imaging using anti-CAIX nanobodies. This mouse model resembles the tumor and tumor micro-environment of human breast cancer more closely than subcutaneous tumor models. The MCF10DCIS cells express little CAIX when grown at normoxic conditions. However, in vivo, these tumor cores can become necrotic and CAIX expression is induced peri-necrotically [11]. Essential for successful translation of optical molecular tracers to a clinically useful tool is a high-imaging contrast. Optimal contrast is established by two factors: high probe accumulation in the tumor in combination with a rapid clearance of the non-bound probe from the body resulting in low background signals. As a direct measure of contrast, we determined the TNR, both in vivo and in an intra-operative setting. Already 1 h after tracer administration, the mean TNR for DCIS + CAIX was ~4.6, which remained stable for the next 8 h. The differences in TNR between DCIS + CAIX and DCIS tumors confirm that, as could be expected, besides the affinity and specificity of the probe, also the expression levels of the molecular target are important to achieve a sufficient TNR. Although CAIX expression is the result of a cellular response to a physiological condition which does not occur in all tumor cells within a tumor, our in vivo studies show that it is sufficient to render tumors fluorescent and detectable with the clinical camera.

The difference between the in vivo and ex vivo TNR can be explained by the limited penetration of light through the skin and subcutaneous tissue, which underscores the notion that optical imaging might especially be suitable for image-guided surgery. As negative control nanobody, we used the non-relevant nanobody R2-IR, which was raised against the molecule RR6 [35]. Our biodistribution studies showed that ~1.9 % of the injected dose of the R2-IR nanobody was found in the tumors, which was clearly higher than found in previous studies where we showed a TNR of approximately 1 [9]. The immunohistochemistry data suggest that this might be caused by a specific binding of this nanobody to vascular tissue surrounding the tumor (Fig. 5j).

An important advantage of the application of nanobodies as targeted probes in optical molecular imaging is the short time interval between probe injection and imaging procedures. The rapid clearance can be related to the detection of B9-IR in the kidneys, as previously described for nanobodies targeting EGFR or HER2 [9, 24]. The previously described hypoxia marker HS680 (*K*_D_ = 8.3 nM; MW 1372) was only rendering tumors visible >6 h post injection [22]. As a direct result of the rapid pharmacokinetics of nanobodies, these probes offer a reduction in logistical burden when applied in a clinical setting, as probe administration and diagnostic or surgical procedures can be performed within a few hours. Further improvement of this system can be expected by the conjugation of several IRDye800CW molecules to the same nanobody, which will require new conjugation strategies.

A promising novel application of molecular imaging in the field of pathology was previously indicated as “molecular fluorescence pathology” [11]. Analysis of tumor sections is normally done with conventional IHC. However, our strategy allows direct analysis of tumors’ molecular status on tissue sections using fluorescence microscopy (Fig. 5). The CAIX-specific nanobody accumulated very well in the perinecrotic areas of the DCIS tumor, where high CAIX expression was confirmed by IHC while low expression was found in surrounding normoxic tumor tissue. As with fluorescence microscopy, various NIR-dyes can be detected at the same time; the problem of heterogenous tumor binding of CAIX-specific nanobodies can be solved by co-injection of two or multiple probes, with specificities for other tumor markers. Also, dual labeling would allow molecular characterization of tumors and could be advantageous for a better delineation of the tumor, which is essential for imaging of tumor margins. Moreover, we anticipate the application of nanobodies in other imaging modalities such as immuno-PET. The rapid clearance of radiolabeled nanobodies allows for application of isotopes with short half-life (Ga-68, half-life 68 min), which will contribute to lower exposure of the patient to radioactive tracers [26, 36]. With this modality, whole body imaging becomes possible, which is very interesting for detection of distant metastases during the course of the breast cancer disease.

## Conclusions

We have produced a novel fluorescent CAIX-specific nanobody and demonstrated the application of this probe preclinically in optical molecular imaging of hypoxic pre-invasive breast cancer before and during surgery. A major advantage of using nanobodies is the high contrast already obtained 2 h after probe administration. Because of these pharmacokinetics, probe injection and surgical procedures can be performed on the same day. Furthermore, the stability of the conjugate allows for “molecular fluorescence pathology”, which might result in better contrast than conventional CAIX-IHC at the pathology department. Molecular fluorescence pathology might be useful for patient-tailored therapeutic decision making in the future. Because of the potentially broad applicability of this probe for many different (hypoxic) tumors, we aim for rapid translation of B9-IR toward clinical studies.

## Electronic Supplementary Material

Below is the link to the electronic supplementary material.

ESM 1 (DOCX 820 kb)
