# Supplementary material for: Hypoxia-Targeting Fluorescent Nanobodies for Optical Molecular Imaging of Pre-Invasive Breast Cancer
Source: Mol Imaging Biol. 2015 Nov 20;18:535–44. doi: 10.1007/s11307-015-0909-6 (PMC4927611; doi:10.1007/s11307-015-0909-6)
Supplement: Supplementary file 1 — (DOCX 820 kb) [file 11307_2015_909_MOESM1_ESM.docx]

# Electronic Supplementary Material

# Hypoxia Targeting Fluorescent Nanobodies for Optical Molecular Imaging of Pre-invasive Breast Cancer

**Journal: Molecular Imaging and Biology**

Aram S.A. van Brussel^1,2^, Arthur Adams^3^, Sabrina Oliveira^1,2^, Bram Dorresteijn^1^, Mohamed El Khattabi^5^, Jeroen F. Vermeulen^2^, Elsken van der Wall^4^, Willem P.Th.M. Mali^3^, Patrick W.B. Derksen^2^, Paul. J. van Diest^2^, and Paul M.P. van Bergen en Henegouwen^1^

Short title: Hypoxia targeting nanobodies for breast cancer imaging

Manuscript category: Article

^1^Division of Cell Biology, Department of Biology, Science Faculty, Utrecht University, Utrecht, The Netherlands

Departments of ^2^Pathology and ^3^Radiology, and ^4^Division of Internal Medicine and Dermatology, University Medical Center Utrecht, Utrecht, The Netherlands

^5^QVQ BV, Utrecht, The Netherlands

### Corresponding author

### Paul M.P. van Bergen en Henegouwen

Division of Cell Biology, Department of Biology, Science Faculty, Utrecht University,

Padualaan 8, 3584 CH, Utrecht, The Netherlands

Phone: +31-30-253 3349; email: [p.vanbergen@uu.nl](mailto:p.vanbergen@uu.nl)

# Material & Methods

*Antibody production, cell lines and cell culture*

MabCAIX antibody was produced as described before [[1](#_ENREF_1)]. A431 epidermoid cancer (CRL-1555), HeLa cervical cancer (CCL-2) and MDA-MB-231 breast cancer cells (HTB-26, ATCC, Wesel, Germany) were grown in Dulbecco’s Modified Eagle’s Medium (DMEM; Invitrogen, Breda, The Netherlands) supplemented with 10% (v/v) Fetal Calf Serum (FCS), 100 IU/ml penicillin, 100 µg/ml streptomycin, and 2 mM L-glutamine, at 37°C in a humidified atmosphere containing 5% CO_2_. MCF10DCIS.com (further referred to as MCF10DCIS) cells (Asterand, Detroit, USA) and MCF-10A cells (CRL-10317, ATCC, Wesel, Germany) were cultured according to the supplier’s guidelines in DMEM/F12, supplemented with 10% (v/v) FCS, 100 IU/ml penicillin, 100 µg/ml streptomycin and 2 mM L-glutamine. Generation of luciferase, and CAIX overexpressing MCF10DCIS cells was performed as described before [[1](#_ENREF_1)]. All cells were cultured at 37°C in a humidified atmosphere containing 5% CO_2_ and were consistently *Mycoplasma* free.

*Cell-based enzyme-linked immunosorbent assay (ELISA)*

A431, MCF-10A, MDA-MB-231 and HeLa cells were seeded in 96-wells plates (15,000 cells per well) and cultured overnight under normoxic (21% O_2_) or hypoxic (2% O_2_) conditions. Next day, cells were put on CO_2_-independent medium supplemented with 4% marvel (dried skimmed milk) and blocked for 30 min at 4^o^C. Next, hypoxic cells were treated with 100 µM deferoxamine (DFO, Sigma Aldrich, Zwijndrecht, The Netherlands). After washing twice with phosphate buffered saline (PBS), cells were incubated with rabbit anti-CAIX antibody (1:2,000; clone ab15086, Abcam, Cambridge, UK) for 1.5h and fixed with 4% paraformaldehyde (PFA) for 30 min. PFA was quenched with NH_4_Cl for 15 min. Primary antibody was detected with donkey anti-rabbit conjugated to peroxidase, after incubation with o-Phenylenediaminedihydrochloride (OPD) with 0.1% H_2_O_2_ used as chromogen and 1 M sulfuric acid was used to stop the peroxidase reaction. Signal was measured using a microplate reader (Bio-Rad model 550, Bio-Rad Laboratories, Veenendaal, The Netherlands).

*Immunization and immune response determination*

Immunizations were approved by the Utrecht University animal ethics committee (DEC#: 2007.III.01.013). For immunization, 8.0 x 10^8^ HeLa cells were cultured under hypoxia (1% O_2_) overnight in an INVIVO2 hypoxia workstation (Ruskinn, Pencoed, UK) and afterwards divided into 8 vials and stored at -80^o^C in medium containing FCS with 10% DMSO and DFO. Two llamas (*Llama glama*) were injected 4 times in the pectoral muscle, with intervals of 2 weeks. Blood was drawn before the first and after the 2^nd^ injection to measure the immune response, and after 8 weeks for construction of the phage library. To measure the immune response, HeLa cells were seeded in two 96-wells plates (1.5 x 10^4^ per well) and grown for 24h under 21% or 1% O_2_. Cells were incubated with serial dilutions of immune or pre-immune llama serum for 2h at 4^o^C. After washing twice with 1% BSA in PBS, cells were fixed in 4% PFA for 20 min, followed by quenching of reactive amine groups with 50mM NH_4_Cl in PBS for 15 min. Bound heavy chain antibodies were detected with anti-VHH polyclonal rabbit serum (1:1,000), and a donkey anti-rabbit antibody coupled to peroxidase (1:5,000). As positive control for hypoxia, MabCAIX was produced as described earlier [[1](#_ENREF_1)], which was detected with a donkey anti-mouse antibody coupled to a peroxidase. OPD with 0.1% peroxide was used as chromogen, and 1 M sulfuric acid was used to stop the peroxidase reaction.

*Library construction*

Construction of phagemid libraries was performed as described before [[2](#_ENREF_2)]. Peripheral B-lymphocytes were isolated from llama blood. RNA was purified by phenol chloroform extraction, and cDNA was generated by reverse transcriptase PCR (SuperScript III, Life Technologies Europe BV, Bleiswijk, The Netherlands). VHH DNA was amplified with PCR using IgG-specific primers and cloned in the pUR8100 phagemid vector. *Escherichia coli* strain TG1 [*sup*E*hsd*_5 *thi* (*lac-proAB*) F(*traD36 proAB_ lacI*q*lacZ_M15*)] was used for the transformation with VHH-phage libraries and for the production of phages.

*Periplasmic fraction production and ELISA screening on cells*

Single *E.coli* TG1 colonies were picked from agar plates and grown in 100 µl 2TY supplemented with 2% glucose and 100 µg/ml ampicillin in sterile 96-wells plates with v-shaped bottom overnight at 37^o^C. Next day, 10 µl of TG1 were used to inoculate 1 ml 2TY supplemented with 100 µg/ml ampicillin in 96-well square V-bottom plates (Corning, New York, USA) for 4h at 37^o^C while shaking. Nanobody production was induced by adding 1mM Isopropyl β-D-1-thiogalactopyranoside (ITPG) and subsequently incubated for 4h at 37^o^C. Bacteria were spun down at 4,600 rpm for 15 min, and pellets were frozen at -20^o^C overnight. Next day pellets were thawed and dissolved in 100 µl PBS. After centrifugation at 4,600 rpm for 15 min the supernatant was used for further screening in an ELISA format. CAIX overexpressing (“DCIS+CAIX”) and non-overexpressing (“DCIS”) MCF10DCIS cells were plated in 96-wells at 12,000 cells per well. Next day, cells were put on CO_2_-independent medium with 4% marvel and blocked for 30 min at 4^o^C. Monoclonal periplasmic fractions (30µl) were added to the wells and incubated for 2h at 4^o^C. After washing twice with PBS, cells were fixed with 4% PFA for 30 min and PFA was quenched with NH_4_Cl/PBS for 15 min. Nanobodies were detected with a rabbit anti-VHH serum and donkey anti-rabbit peroxidase. OPD with 0.1% H_2_O_2_ was used as chromogen.

*Nanobody production and purification*

Nanobodies were re-cloned from the pUR8100 phagemid vector into the pQVQ72 expression vector (kindly provided by QVQ BV, Utrecht, The Netherlands), which introduces a C-terminal cysteine, flanked by a Flag-tag to enable site-directed conjugation of IRDye800CW-Maleimide (LI-COR Biosciences, Lincoln, NE). After transformation into *E.coli* TG1, 800 ml cultures were grown in 2x Tryptone Yeast Extract Medium (2TY), until optical density of 0.5 at 600 nm (OD600: 0.5) was reached. Nanobody production was induced by adding 1 mM IPTG and 4h after induction bacteria were harvested by centrifugation. Pellets were frozen at -20^o^C and next day the periplasmic fraction was obtained by thawing, resuspension in PBS, followed by spinning for 2h head over head at 4^o^C, and centrifugation at 4,600 rpm for 15 min. Nanobodies were purified from the periplasmic fraction by affinity chromatography using a HiTrap protein A HP column (GE Healthcare, Zeist, The Netherlands) using the ÄKTAxpress system (GE Healthcare, Zeist, The Netherlands).

*Immunofluorescence*
Cover glasses were coated with 0.25% gelatin and seeded with co-cultures of 2.0*10^4^ CAIX-overexpressing and CAIX negative MCF10DCIS cells. Cells were incubated with 1 µM nanobodies for 2h at 4^o^C. After washing twice with PBS, cells were fixed in 4% PFA for 30 min and quenched with 100 mM glycin in PBS for 10 min. Nanobodies were detected with a rabbit anti-VHH at 1:500 and CAIX-FLAG was detected with mouse anti-FLAG M2 (1:500, Sigma Aldrich, Zwijndrecht, The Netherlands). Subsequently, a goat anti-rabbit Alexa488 and a goat anti-mouse Alexa555 (Life Technologies Europe BV, Bleiswijk, The Netherlands) were used for detection, using a confocal microscope (Zeiss, Sliedrecht, The Netherlands) .

For competition studies, cover glasses were seeded with either CAIX-overexpressing DCIS+CAIX cells or CAIX negative DCIS cells. Cells were cooled to 4^o^C and blocked using 1% BSA in culture medium for 30 min at 4°C. Cells were then incubated with 2.5 nM of the B9 nanobody for 2h at 4^o^C. In parallel, cells were first pre-incubated with a mixture of 2.5 nM B9 and 500-fold molar excess of recombinant human CAIX (rh CAIX, R&D systems, #2188-CA, Minneapolis, USA) for 15 min at RT, followed by the 2h incubation at 4^o^C. Negative controls were incubated with either rhCAIX or PBS alone. After washing twice with cold PBS, cells were fixed with 4% PFA for 30 min and quenched with 100 mM glycin/PBS for 10 min. The nanobody B9 was detected with a rabbit anti-VHH at 1:500 dilution. Subsequently, a goat anti-rabbit Alexa488 antibody was used for detection followed by 10 min incubation with DAPI. Cells were visualized using a LSM700 confocal microscope (Zeiss, Sliedrecht, The Netherlands).

*Binding affinity determination*

For determination of binding affinity 2.0*10^4^ DCIS+CAIX cells overexpressing CAIX were seeded per well one day in advance and incubated at 4^o^C for 1.5h with dilution series of the nanobodies in PBS in triplicate. The detection of bound nanobodies was performed using the rabbit anti-VHH serum, followed by a donkey anti-rabbit peroxidase antibody. OPD with 0.1% H_2_O_2_ was used as chromogen. The dissociation constant (K_D_) was derived from the concentration of nanobodies at which half the intensity of B_max_ was found using non-linear regression, of one site specific binding, (Graphpad Prism v.5, GraphPad Software, La Jolla, USA).

*Conjugation of IRDye800CW to CAIX nanobodies*

Before IRDye800CW labeling, nanobodies were reduced by adding 70-fold molar excess of Tris (2-CarboxyEthyl) Phosphine hydrochloride (TCEP). IRDye800CW-Maleimide (further referred to as IR; LI-COR) was conjugated to nanobodies following manufacturer recommendations, with molar dye to protein ratios of 4:1. After labeling, free dye was separated from the conjugate using two sequential Pierce Zeba^TM^ Desalting Spin Columns (Thermo Fisher Scientific, Landsmeer, The Netherlands). The degree of labeling (DOL) was calculated using the absorbance at 280 nm (A_280_) and 774 nm (A_774_) with a NanoDrop spectrophotometer (NanoDrop Technologies, Wilmington, Delaware, USA). DOL was calculated with the formula: dye/protein=( A_774_/ε_dye_) / (A_280_-(0.03 * A_774_) / ε_prot_), where the molar extinction coefficient of IRDye800CW (ε_dye_) is 240,000 M^-1^ cm^-1^ and the molar extinction coefficients for the protein (ε_prot_) is 31,400; 25,900; 31,400; 31,400 and 30,940 M^-1^ cm^-1^ for CAIX1, E4, B9, C5 and R2 nanobodies respectively.

*Characterization of IR-conjugated nanobodies*

IR-conjugated proteins (1 µg) were size-separated with 15% SDS-PAGE. Gels were stained with Coomassie Brilliant Blue solution (SERVA Electrophoresis GmbH, Heidelberg, Germany) and imaged with the Odyssey imaging system (LI-COR Biosciences, Lincoln, NE) using the 700 nm channel for the Coomassie stain and the 800 nm channel for IR detection. For affinity determination of IR-labelled nanobodies, 2.0*10^4^ MCF10DCIS cells overexpressing CAIX were seeded per well, one day in advance and incubated at 4^o^C for 1.5h with dilution series of IR-labelled nanobody in PBS in triplicate. The detection of bound IR-labelled nanobody was performed using the 800 nm channel of the Odyssey imaging system. The dissociation constant (K_D_) was derived from the concentration of nanobodies at which half the intensity of B_max_ was found using non-linear regression, of one site specific binding, (Graphpad Prism v.5, GraphPad Software, La Jolla, USA).

*In vivo experiments*

All animal experiments were approved by the Utrecht University Animal Experimental Committee (DEC#. 2012.III.02.015). The mouse model used in this study was based on a previously described model [[3](#_ENREF_3), [4](#_ENREF_4)]. A real-time intra-operative multispectral fluorescence reflectance imaging (MFRI) system (SurgOptix, Groningen, The Netherlands) was used to monitor uptake and washout of the fluorescent nanobody over time as described before [[1](#_ENREF_1), [4](#_ENREF_4), [5](#_ENREF_5)]. TNR analysis was performed using GraphPad Prism (v.5, GraphPad Software). TNRs, obtained during optimal imaging time points after injections with B9 nanobody or non-relevant R2 nanobody, were compared using the Mann-Whitney U test (two-tailed).

*Imaging of fluorescent sections and immunohistochemistry*

Immunohistochemistry (IHC) for CAIX and haematoxylin and eosin (H&E) stainings were performed as described before [[4](#_ENREF_4)]. Immediately after resection (3h post injection), tumors were fixed in neutral buffered formalin, routinely processed to paraffin blocks and stored in the dark until further processing. Following deparaffination and rehydration, 4 µm thick sections were mounted in Vectashield Mounting Medium (Vector Laboratories, Burlingame, CA, USA) and scanned using the Odyssey imaging system at highest (21 µm) resolution and highest quality. Immunohistochemistry (IHC) for CAIX and haematoxylin and eosin (H&E) stainings were performed as described before [[4](#_ENREF_4)]. Slides were scanned with the Scanscope XT 120 scanner (Aperio, Vista, CA, USA).

*Biodistribution*

Quantification of IR-conjugated nanobodies in tumors and tissues was done as previously described [[6-8](#_ENREF_6)]. Tumors and organs collected 3h after probe injection were weighed, snap-frozen in liquid nitrogen and stored at -80^o^C until further processing. After homogenization and lysis of the organs, dilution series, including pre-defined concentrations of nanobody-IR for calibration, were made in Nunc 96-well plates (Thermo Fisher Scientific, Landsmeer, The Netherlands) with PBS and scanned with the Odyssey imaging system. Thereafter, the quantity of each probe in each organ (in percentage of injected dose per gram of tissue) was determined by intra- or extrapolation of the unknown fluorescence values from the respective calibration curves using the GraphPad Prism software (v.5, GraphPad Software, La Jolla, CA, USA).

**Results**

The specific binding of the selected anti-CAIX nanobody B9, was investigated by comparing the binding of B9 to either CAIX-overexpressing DCIS+CAIX cells or the CAIX negative DCIS cells under normoxic conditions. Secondly, a competition experiment was performed using the purified ectodomain of CAIX as competitor for B9 binding. A non-saturating concentration of the B9 nanobody was shown to bind DCIS+CAIX cells (Fig. 1S). A 500-fold molar excess of rhCAIX completely blocked the binding of B9 to DCIS+CAIX cells. These data demonstrate the specificity of the selected anti-CAIX nanobody B9


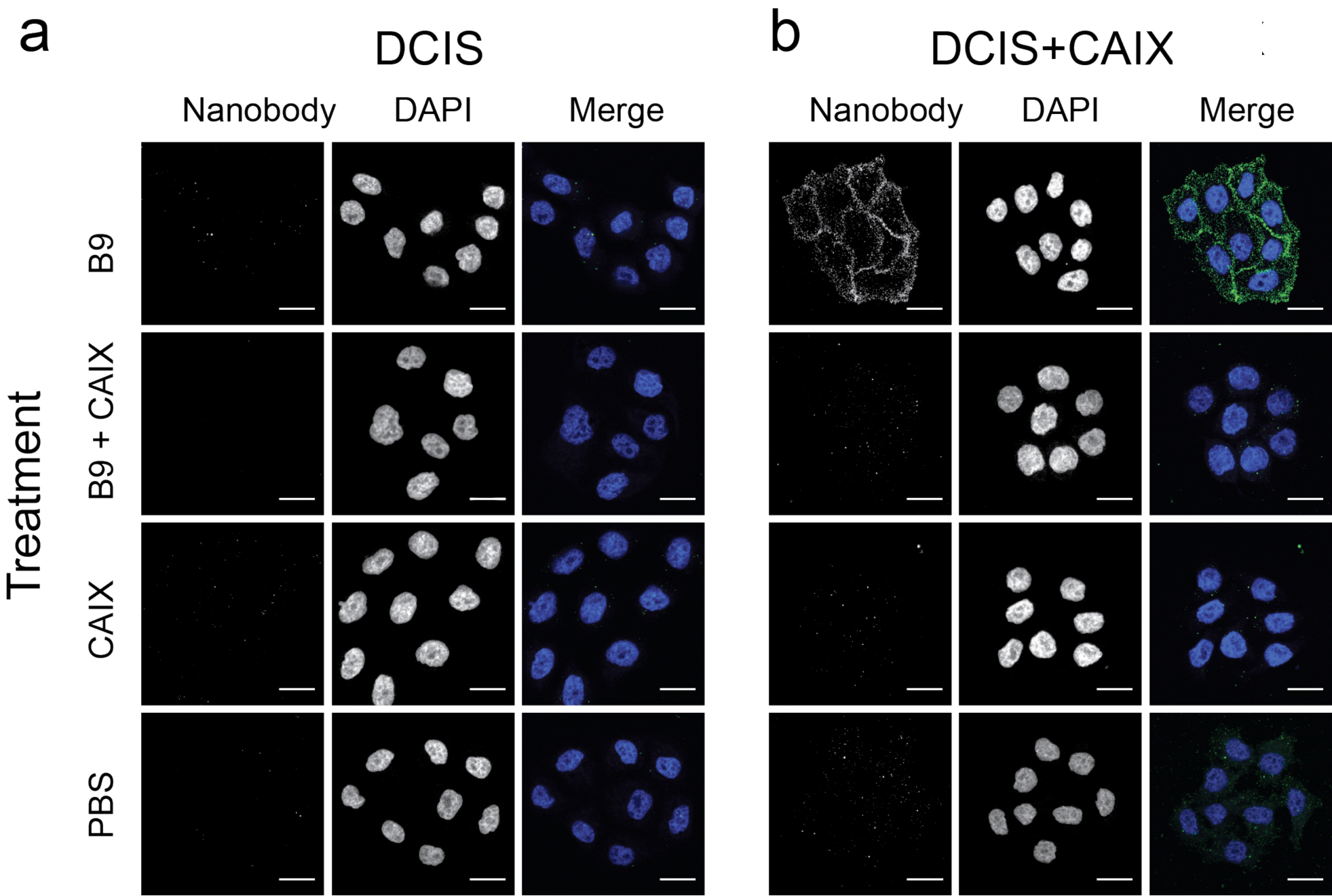


Figure 1S. **Specificity of the B9 nanobody for CAIX.** DCIS cells with no expression of CAIX (a) and DCIS+CAIX cells ectopically expressing CAIX (b) were incubated with 2.5 nM B9 (B9), a 500-fold molar excess of recombinant human CAIX (CAIX), a combination of both (B9 + CAIX) or left untreated (PBS). Bound B9 nanobody was detected with rabbit anti-VHH and goat anti-rabbit Alexa488 antibodies. After counterstaining with DAPI cells were imaged by confocal fluorescence microscopy. Scale bar 20 µm.

**References**

1. van Brussel AS, Adams A, Vermeulen JF*, et al.* Molecular imaging with a fluorescent antibody targeting carbonic anhydrase IX can successfully detect hypoxic ductal carcinoma in situ of the breast. *Breast Cancer Res Treat* 2013.

2. Roovers RC, Laeremans T, Huang L*, et al.* Efficient inhibition of EGFR signaling and of tumour growth by antagonistic anti-EFGR Nanobodies. *Cancer Immunol Immunother* 2007; **56**: 303-317.

3. Behbod F, Kittrell FS, LaMarca H*, et al.* An intraductal human-in-mouse transplantation model mimics the subtypes of ductal carcinoma in situ. *Breast Cancer Res* 2009; **11**: R66.

4. Vermeulen JF, van Brussel AS, Adams A*, et al.* Near-Infrared Fluorescence Molecular Imaging of Ductal Carcinoma In Situ with CD44v6-Specific Antibodies in Mice: A Preclinical Study. *Mol Imaging Biol* 2013; **15**: 290-298.

5. Themelis G, Yoo JS, Soh KS*, et al.* Real-time intraoperative fluorescence imaging system using light-absorption correction. *J Biomed Opt* 2009; **14**: 064012.

6. Oliveira S, van Dongen GA, Stigter-van Walsum M*, et al.* Rapid visualization of human tumor xenografts through optical imaging with a near-infrared fluorescent anti-epidermal growth factor receptor nanobody. *Mol Imaging* 2012; **11**: 33-46.

7. Kijanka M, Warnders FJ, El Khattabi M*, et al.* Rapid optical imaging of human breast tumour xenografts using anti-HER2 VHHs site-directly conjugated to IRDye 800CW for image-guided surgery. *Eur J Nucl Med Mol Imaging* 2013; **40**: 1718-1729.

8. Oliveira S, Cohen R, Walsum MS*, et al.* A novel method to quantify IRDye800CW fluorescent antibody probes ex vivo in tissue distribution studies. *EJNMMI Res* 2012; **2**: 50.
